# Hybrid Recanalization for the Treatment of Carotid/Vertebral In-stent Restenosis or Occlusion: Pilot Surgery Experiences From One Single Center

**DOI:** 10.3389/fneur.2020.604672

**Published:** 2020-11-24

**Authors:** Chao Wang, Peng Zhao, Tao Sun, Mengtao Han, Yunyan Wang, Wei Wu, Xingang Li, Donghai Wang

**Affiliations:** ^1^School of Clinical Medicine, Shandong University, Jinan, China; ^2^Dezhou City People's Hospital, Dezhou, China; ^3^Department of Neurosurgery, Binzhou Medical University Hospital, Binzhou, China; ^4^Department of Neurosurgery, Qilu Hospital of Shandong University and Institute of Brain and Brain-Inspired Science, Shandong University, Jinan, China; ^5^Department of Neurology, Qilu Hospital of Shandong University and Institute of Brain and Brain-Inspired Science, Shandong University, Jinan, China

**Keywords:** endarterectomy, hybrid recanalization, occlusion, restenosis, stent

## Abstract

**Background :** The hybrid recanalization of internal carotid artery (ICA) and vertebral artery (VA) in-stent restenosis or occlusion using a combination of endarterectomy and endovascular intervention has achieved technical success. We present our surgical experiences to further evaluate the safety and efficacy of the hybrid technique for the treatment of in-stent restenosis and occlusion.

**Methods :** A cohort of 12 refractory patients with in-stent restenosis or occlusion who underwent hybrid recanalization, a combination of endarterectomy and endovascular intervention, were retrospectively analyzed. Medical records, including presenting symptoms, comorbidities, contralateral ICA/VA findings, use of antiplatelet drugs, postoperative complications, and angiographic outcomes, were collected.

**Results :** Among 415 consecutive patients with ICA, common carotid artery, and V1 segment lesions, 12 refractory patients (2.89%) with 13 cases were enrolled in our study (1 female and 11 male). All patients underwent successful hybrid recanalization. There were no cases of postoperative stroke or death. Only two patients sustained hoarseness, but it resolved within 2 weeks after surgery. Three patients were treated with dual antiplatelet (aspirin and clopidogrel), seven with single antiplatelet (aspirin), one with single antiplatelet (clopidogrel), and one with single antiplatelet (ticagrelor). All patients were followed up in the outpatient department according to the protocol, with a mean follow-up period of 13 months (range, 6–24 months). No death or recurrent symptoms occurred during the regular follow-up period.

**Conclusion :** The hybrid technique maybe a safe and feasible treatment option to recanalize in-stent restenosis or occlusion with acceptable complications.

## Introduction

Stroke derived from carotid or vertebral artery stenosis or occlusion poses a major problem worldwide and is associated with a high risk of recurrence and poor outcome. Although stent implantation in the carotid or vertebral stenosis has been acknowledged as a safe option for reducing long term stroke risk, high rates of stroke recurrence due to in-stent restenosis (ISR) and occlusion (ISO) after carotid artery stenting (CAS; 5–11%) or vertebral artery stenting (VAS; 18–43%) have been reported (1–4). Different treatment strategies, including pharmacological therapy, endovascular therapy, and surgery, are applied for the revascularization of ISR or ISO; however, no optimal therapeutic approach is recommended at present (2, 5). To our knowledge, hybrid techniques are less utilized mainly because of the high demand for hybrid operation rooms and neurosurgeons (6, 7). Moreover, previous studies of hybrid revascularization are mainly limited to an article on the chronically occluded internal carotid artery and to case reports of VA ISO or ICA ISR (3, 6, 8, 9). Therefore, in this single-center study, the pilot experiences of hybrid recanalization in 12 patients with 13 cases of carotid/vertebral ISR or ISO were reported.

## Methods

### Patients

From October 2016 to June 2020, 415 consecutive patients with internal carotid artery (ICA) and V1 segment stenosis or occlusion were admitted to our center for ischemic stroke. Written informed consent was obtained from the individuals for the publication of any potentially identifiable images or data included in this article. All patients underwent digital subtraction angiography (DSA) to confirm the lesion. Medical treatment including single antiplatelet therapy (aspirin 100 mg/d, clopidogrel 75 mg/d, or ticagrelor 180 mg/d for 90 days) plus statin and the management of risk factors (e.g., hypertension, diabetes, and lifestyle) was performed. In this study, patients were included if they met the following criteria: (1) ISR (≥70%) or occlusion of the ICA, the common carotid artery (CCA), or the V1 verified by DSA; (2) recurrent strokes or transient ischemic attacks (TIA) refractory to best medical treatment; (3) primary treatment performed using stent angioplasty; (4) available clinical and angiographic follow-up data after primary treatment and after hybrid recanalization for ISR or ISO. Exclusion criteria were: (1) lack of clinical or imaging information and (2) primary treatment only performed using balloon angioplasty or endarterectomy.

### Hybrid Revascularization Technique

Under general anesthesia, transfemoral access was performed first. Then, a standard carotid endarterectomy (CEA) or vertebral endarterectomy (VEA) surgery was performed to remove the plaque with the stent. After endarterectomy, a continuous suture was performed if there was retrograde blood flow. In case of no bleeding, a Fogarty balloon (4F for CEA/3F for VEA) thrombectomy was performed through the exposed proximal ICA or V1 segment to remove the distal thrombus. Then, a balloon-expandable (for VA) or self-expanding (for ICA and CCA) stent was implanted if there was distal stenosis or dissection through rechecked angiography. Finally, angiography was performed to ensure vascular patency. Heparin anticoagulation therapy was maintained during the temporary clamping and endovascular procedure. Blood pressure was tightly controlled postoperatively. Patients were prescribed single antiplatelet treatment after endarterectomy or balloon thrombectomy, and dual antiplatelet medications after additional stent implantation.

### Clinical and Imaging Follow-Up

Clinical follow-up was performed by telephone interview or clinic visits at 1, 3, and 6 months after the intervention and yearly thereafter. Clinical events including TIA, stroke (of both anterior and posterior circulation), myocardial infarction, and death were recorded as clinical outcomes. Postoperative CT angiography (CTA) follow-up was mostly performed at 3 and 6 months after the intervention and then yearly. DSA and magnetic resonance imaging (MRI) assessment was performed according to the decision of the physicians once the patient presented with recurrent stroke or TIA.

## Results

### Clinical Characteristics

Of 415 consecutive patients with ICA, CCA, and V1 segment lesions, 12 refractory patients (2.89%) were enrolled in our study (1 female and 11 male). The mean age was 65 years (range, 44–79 years). Comorbidities included tobacco smoking in 4 (33.33%) patients, hypertension in 10 (83.33%), hyperlipidemia in 1 (8.33%), coronary heart disease in 2 (16.67%), and diabetes mellitus in 4 (33.33%). All patients had recurrent TIA despite aggressive medical therapy (antiplatelet, statin, antihypertensive, etc.). Of the 12 patients, 7 had CAS-ISR, 1 had CAS-ISO, 3 had VAS-ISO, and 1 had concomitant CAS-ISR and VAS-ISO.

### Surgery, Antiplatelet Therapy, and Follow-Up Evaluation

All patients underwent successful hybrid recanalization. Among them, two patients underwent balloon thrombectomy and three had additional stent implantation. There were no cases of postoperative stroke or death. Only two patients sustained hoarseness, but it resolved within 2 weeks after surgery. All patients underwent thromboelastography platelet mapping analysis, and ticagrelor was used as an alternative if the test indicated no response to a dual antiplatelet regimen. As a result, three patients were treated with dual antiplatelet (aspirin and clopidogrel), seven with single antiplatelet (aspirin), one with single antiplatelet (clopidogrel), and one with single antiplatelet (ticagrelor). All patients were followed-up in the outpatient department according to the protocol, with a mean follow-up period of 13 months (range, 6–24 months). Only two patients showed a low grade stenosis (<30%), and the rest remained a normal luminal diameter after rechecking the latest imaging (CTA) follow-up. No death or recurrent symptoms occurred during the regular follow-up period. The clinical data and outcomes are summarized in Table 1.

**Table 1:** Baseline characteristics, clinical data, and outcomes.

**No**	**Sex/age (years)**	**Recurrent symptoms**	**Smoking history**	**Hypertension**	**Hyperlipidaemia**	**Coronary heart disease**	**Diabetes**	**ISR/ISO type**	**Period from stenting to recurrence (month)**	**Contralateral ICA/VA**	**Additional endovascular device**	**Postoperative complications**	**Plaque topography and composition**	**Antiplatelet drugs**	**CTA follow-up**	**Follow-up time (month)**
1	M/60	Limb weakness, slurred speech	–	+	–	–	–	CAS-ISO	30	ICA occlusion	Balloon, stent	None	Concentric, long-segmental	Aspirin, clopidogrel	Normal	6
2	M/66	Limb numbness	+	+	–	–	+	CAS-ISR	8	ICA moderate stenosis	None	None	Eccentric, proximal	Aspirin	Normal	12
3	M/79	Limb weakness	–	+	–	–	–	CAS-ISR	14	Normal	None	None	Eccentric, proximal	Aspirin	Low grade stenosis	16
4	M/70	Limb weakness, syncope	–	+	–	–	–	CAS-ISR	26	Normal	None	None	Eccentric, proximal	Clopidogrel	Normal	24
5	F/70	Limb numbness, slurred speech	–	–	–	–	–	CAS-ISR	5	Normal	None	None	Eccentric, proximal	Aspirin	Low grade stenosis	24
6	M/62	Limb weakness, slurred speech	–	+	–	–	+	CAS-ISR	7	Normal	None	None	Eccentric,proximal, stent fracture	Ticagrelor	Normal	6
7	M/67	Limb numbness	+	+	–	+	+	CAS-ISR	19	Normal	None	None	Eccentric, proximal	Aspirin	Normal	6
8	M/69	Limb weakness	–	–	–	–	–	CAS-ISR	10	Normal	None	None	Eccentric, proximal	Aspirin	Normal	6
9	M/54	Dizziness, walking instability	+	+	–	–	–	VAS-ISO	5	VA dominance, PICA deficiency	None	Hoarseness	Concentric, long-segmental	Aspirin	Normal	22
10	M/65	Dizziness, slurred speech	–	+	–	–	–	VAS-ISO	5	Normal	None	None	Concentric, long-segmental	Aspirin	Normal	12
11	M/70	Dizziness	–	+	–	–	–	VAS-ISO	10	V4 mild stenosis	Stent	None	Concentric, long-segmental	Aspirin, clopidogrel	Normal	12
12	M/44	Transient dizziness, limb numbness	+	+	+	+	+	CAS-ISR, anitha VAS-ISO	3	Normal	Balloon, stent	Hoarseness	CAS- Eccentric, proximal; anitha VAS- Concentric, long-segmental	Aspirin, clopidogrel	Normal	6

*CAS, carotid artery stenting; ICA, internal carotid artery; ISO, in-stent occlusion; ISR, in-stent restenosis; VAS, vertebral artery stenting; VA, vertebral artery*.

### Topography and Composition of the Plaque

Postoperative plaque topography (eccentric/concentric, proximal/long-segmental lesion) and morphology (stent fracture) were recorded. Plaque topography revealed eccentric plaques in eight (66.67%) CAS cases and proximal plaques in eight (66.67%) CAS cases. However, VAS cases were more prone to concentric and long-segmental lesions. Plaque morphology revealed stent fracture in one (8.33%) CAS case.

### Representative Case Illustrations

#### Patient 1

**Figure 1:**
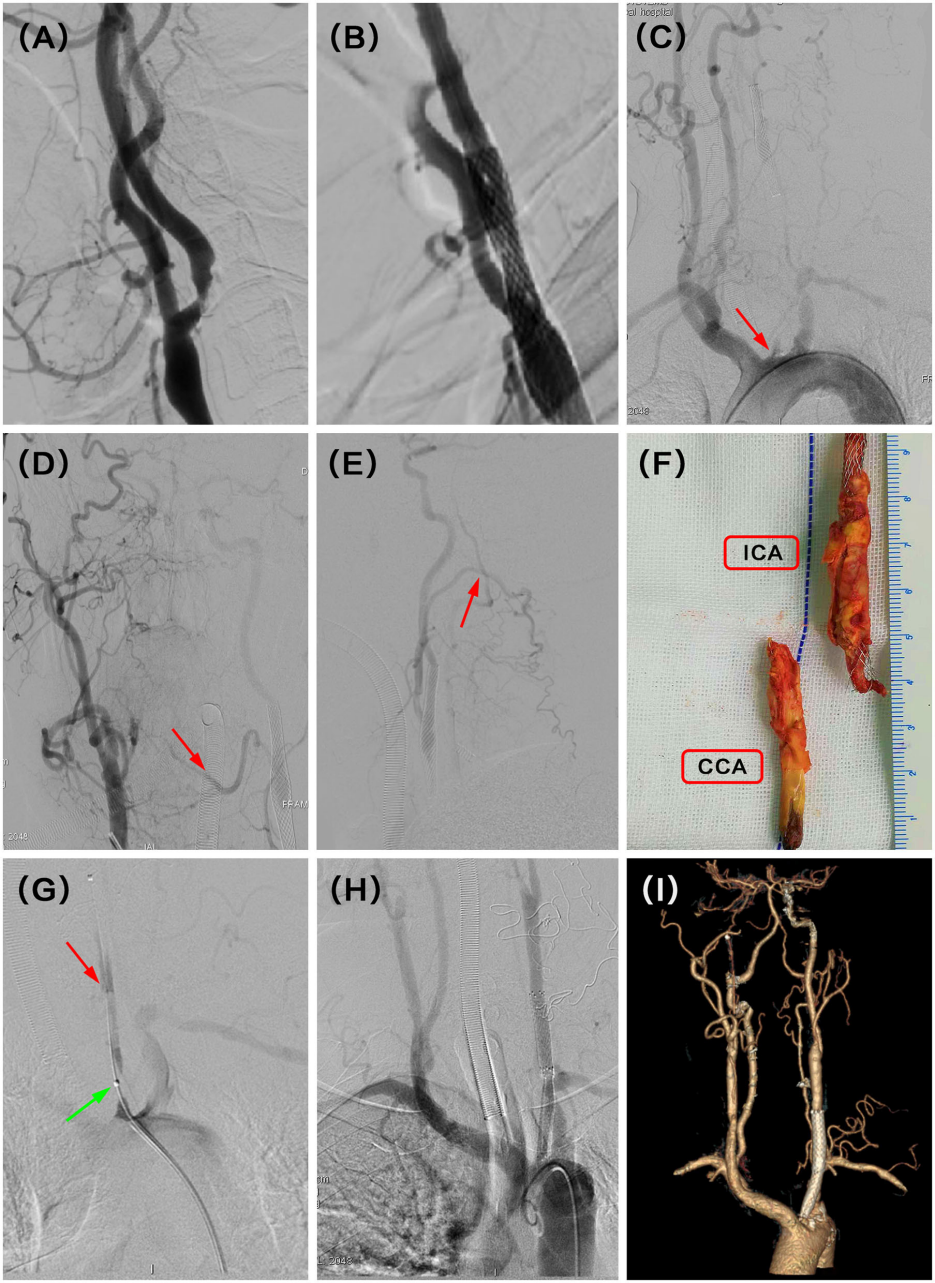
**(A)** Severe stenosis of the left proximal internal carotid artery (ICA) was observed in the digital subtraction angiography (DSA). **(B)** Revascularization was achieved with balloon and stent angioplasty. **(C)** DSA showed recurrent left proximal common carotid artery (CCA) occlusion at the 30 months follow-up (red arrow). **(D)** Intraoperative angiography demonstrated that complementary inflow was reconstituted by collaterals from right lingual branches (red arrow). **(E)** Another complementary inflow was also reconstituted by collaterals from left occipital branches (red arrow). **(F)** Carotid endarterectomy (CEA) combined with Fogarty balloon thrombectomy was performed to remove the plaque with a stent, and the stent was seen clearly in the gross specimen. **(G)** After that, intraoperative angiography in the beginning of CCA showed tandem severe stenosis (green arrow) and dissection (red arrow) at the proximal and median CCA, respectively. **(H)** Successful recanalization was then achieved with further stent angioplasty with balloon dilation. **(I)** CT angiography (CTA) indicated the patency of the left CCA at the latest follow-up time (6 months) after hybrid surgery.

A 60-year-old man presented with dizziness. He additionally demonstrated paroxysmal limb weakness and slurred speech despite the best pharmacological option. DSA demonstrated occlusion of the left CCA 30 months after CAS (Figures 1A–C). CCA was reconstituted by collaterals from the contralateral lingual and ipsilateral occipital branches (Figures 1D,E). He underwent successful hybrid recanalization for the left CCA and the stent was clearly seen in the gross specimen (Figures 1F–H), and the 6-month postoperative CTA showed persistent patency (Figure 1I).

#### Patient 9

**Figure 2:**
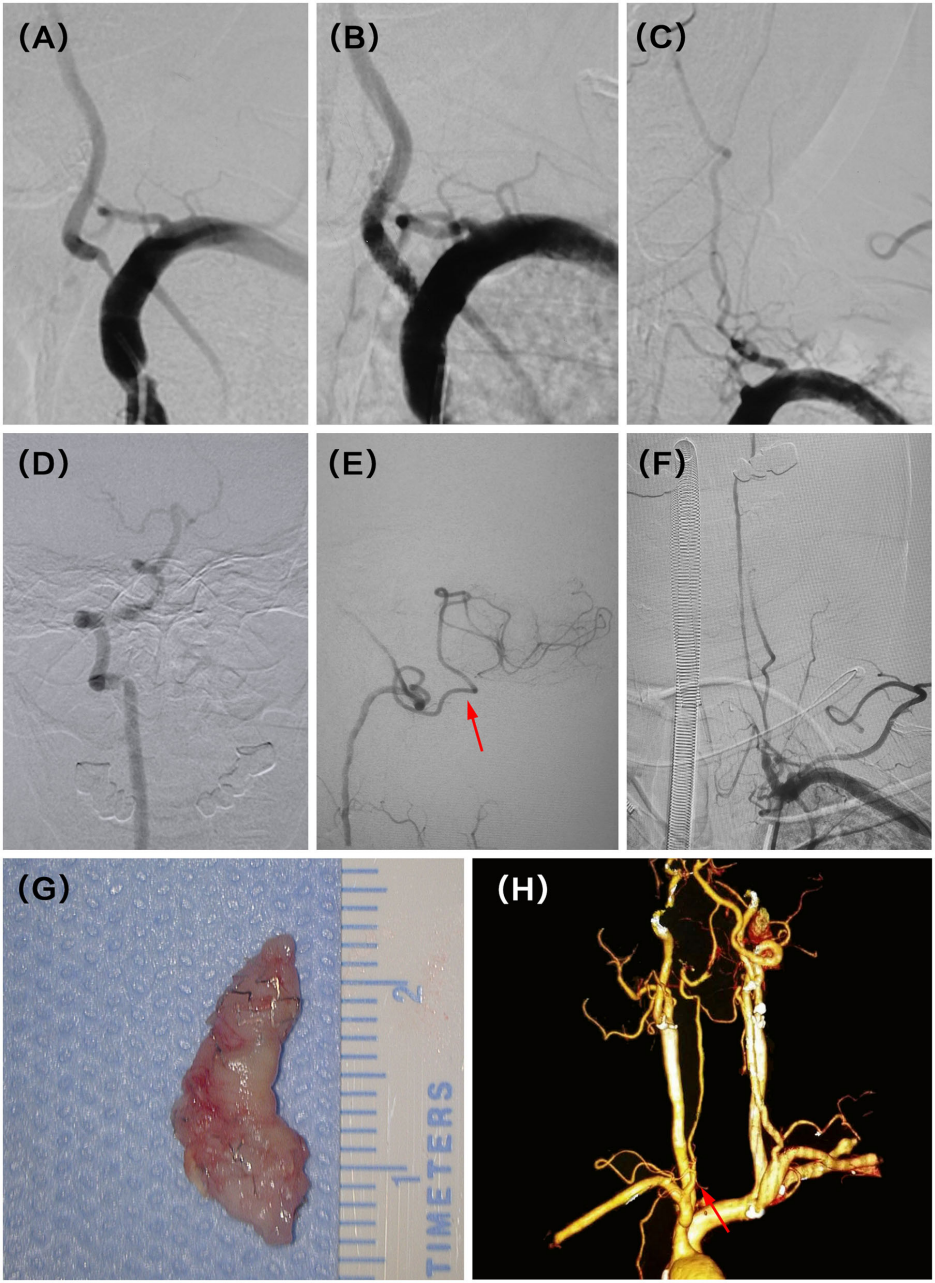
**(A)** Severe stenosis of the left vertebral artery (VA) ostium was observed in the digital subtraction angiography (DSA). **(B)** Revascularization was achieved with balloon and stent angioplasty. **(C)** DSA showed recurrent left V1 occlusion at the 5 months follow-up. **(D)** Intraoperative angiography demonstrated dominant right VA with the deficiency of ipsilateral PICA. **(E)** Intraoperative angiography also demonstrated symptomatic non-dominant left VA with a thin posterior inferior cerebellar artery (PICA) (red arrow). **(F)** Successful recanalization was then achieved with stent removal after vertebral endarterectomy (VEA). **(G)** The stent was seen clearly in the gross specimen. **(H)** CT angiography (CTA) indicated the patency of the left VA at the latest follow-up time (22 months) after hybrid surgery (red arrow).

A 54-year-old man presented with recurrent dizziness. He later developed walking instability. DSA demonstrated left V1 occlusion 5 months after stenting (Figures 2A,B). V1 was reconstituted by collaterals from muscular and thyrocervical branches (Figure 2C). Although the right VA was dominant, the deficiency of the right posterior inferior cerebellar artery (PICA) and hypoperfusion of the left PICA resulted in recurrent and aggravating symptoms (Figures 2D,E). He underwent hybrid recanalization for the left VA and the stent was clearly seen in the gross specimen (Figures 2F,G), and the 22-month postoperative CTA showed persistent patency (Figure 2H).

## Discussion

The rates of ischemic cerebrovascular events involving the carotid bifurcations or the vertebro-basilar circulation have been estimated at 7–20% and 25%, respectively (10–13); these are considered high rates. Stenting is regarded as the preferred option for carotid and vertebral artery stenosis surgery for its low invasiveness, low patient discomfort, and short hospitalization time, among other reasons (14, 15). However, the widespread use of CAS and VAS leads to the occurrence of early and late complications, like ISR or ISO. The benefits of stenting in the prevention of future stroke may be hampered by the high rate of ISR or ISO (2, 16). In our study, we presented a hybrid recanalization treatment in 12 patients with carotid or vertebral ISR/ISO with good follow-up results. In addition, the illustrative cases demonstrate the challenges that a neurosurgeon or neurointerventionalist can face when managing such cases.

ISR has been defined by an angiography cutoff value of ≥50 or ≥70% in several studies (3, 5, 15). We used the latter as our standard in all cases after angiographic calculation. Duplex ultrasonography also correlated very well with catheter-based angiography. Peak systolic velocity (PSV) of 300 cm/s, end-diastolic velocity (EDV) of 140 cm/s, and ICA/CCA of 3.8 are, as well, optimal cutoff points to detect 70% restenosis after stenting (17, 18).

With respect to the pathogenesis of this process, early ISR (<6 weeks) is caused by myointimal hyperplasia and vascular remodeling, and late ISR is a consequence of recurrent neo-atherosclerosis (19, 20). Compared with VA plaques, ICA plaques were more prone to eccentric lesions; this was largely due to less shear stress on the lateral wall, where most plaques occurred (21, 22). Stent fracture has also been previously reported to predict plaque formation due to abnormal endothelial proliferation caused by mechanical disruption of the fracture. An incidence of stent fracture up to 4–25% has been reported in previous studies, but the stent fracture incidence in our cohort was lower, probably due to the specific inclusion criteria (23).

The risk factors for ISR may originate from three sources: the patients, physician skill, and stent characteristics (5, 24, 25). The first source includes factors such as smoking, hypertension, hyperlipidemia, atrial fibrillation, diabetes mellitus, C-reactive protein (>5 mg/l), and coronary artery disease. These could be controlled by pharmacological and lifestyle management (5, 24). The second source includes high postoperative residual stenosis caused by the lack of skill of the surgeon. A high degree of residual stenosis with a corresponding small vascular diameter is an independent risk factor for ISR, and technical success is mostly defined as residual stenosis <30% (24). Finally, the third source includes a small stent diameter, which is consistent with a small artery size and has been reported as another predictor of ISR (25).

Standard pharmacological management of ISR-including antiplatelet, statin, and antihypertensive drugs-is the first step before further treatment (2, 5). The efficacy of medication in reducing the long-term stroke risk remains controversial. The Vertebral artery Ischemic Stenting Trial (VIST) showed that pharmacological treatment was preferred in cases of intracranial vertebral stenosis because of high operative risk; the Vertebral Artery Stenting Trial (VAST) demonstrated a higher same territory stroke risk in the stented group than in the pharmacological treatment group (26, 27). However, the Warfarin–Aspirin Symptomatic Intracranial Disease (WASID) study group concluded that many patients with symptomatic VA ostitum disease continued to have ischemic events despite optimal pharmacological therapy. A previous study also demonstrated that recurrent symptoms still appeared in patients treated with maximal pharmacological therapy, and 18% of the ICA stenosed patients developed a severe disability or died during the follow-up (28, 29).

Endovascular treatment mainly included balloon or stent angioplasty. Balloon is considered preferable to stent angioplasty because of its higher patency rates during short-term follow-up and because of the challenge of passing the catheter through the in-stent restenosis (5). However, multiple studies have demonstrated a trend toward choosing stenting over balloon angioplasty for its lower restenosis rates during long-term follow-up (30, 31). Different types of the stent also resulted in different ISR rates. A prospective randomized trial reported a lower ISR rate of 3.1% in the self-expanding stent group compared with 22.9% in the balloon-expandable stent group; the favorable outcome of the self-expanding stent is likely due to its flexibility and compliance to the tortuous vessels. Previous studies have also shown that drug-eluting stents were associated with a lower restenosis rate (11%) than were bare metal stents (19.4–30%) due to their ability to inhibit endothelial proliferation (32, 33).

Surgical revascularization includes primary endarterectomy, transposition, and a hybrid technique. CEA is a conventional treatment that can remove plaque with a stent, while VEA is not widely used due to its technical challenge and the lack of supporting findings from randomized trials (6, 7). VA transposition is a good alternative treatment for proximal VA occlusive disease, but it is difficult to achieve recanalization of the distal VA (1). Therefore, a hybrid technique that combines endarterectomy and endovascular intervention has emerged as an alternative procedure to achieve recanalization, especially for long-segmental occlusion. Open surgery could not only obtain proximal revascularization but also make the real lumen (the interface between the plaque and vessel wall) visible (7, 34). A technique reported in previous studies was the insertion of a microwire into the real lumen of the VA to ensure the completion of subsequent thrombectomy and stenting (6, 35). However, considering the mostly straight anatomy of the proximal ICA and the V1 segment and the performance of preoperative angiography evaluation, the Fogarty balloon was directly inserted through the exposed proximal ICA or V1 segment to conduct the surgical thrombectomy in our center. In addition, complementary endovascular thrombectomy or stenting could also achieve distal recanalization and avoid destroying the collaterals from the thyrocervical or costocervical trunk (35). In addition to wound hematomas and infections, Horner syndrome and chylothorax are inherent risks of the process of VA endarterectomy and transposition (1, 7). In our study, two patients in the VAS group developed transient nerve dysfunction after surgery, whereas none of the CAS group developed this complication. There were two main reasons for this disparity. First, the endarterectomy required a delicate operative maneuver under the microscope. Second, the operation area of CEA was always limited to the carotid sheath after it was cut open, observing little injury to the cranial nerve. In addition, the surgical ligation of the left thoracic duct was performed to prevent postoperative chylothorax in VA hybrid recanalization.

This study had several limitations. To begin with, it was a retrospective, small sample size, single-center design, and one hybrid technique study. Furthermore, a longer and sufficient follow-up including cranial MRI, is needed to evaluate neurologic improvement, the patency of the recanalized ICA or VA, and the efficacy of hybrid surgery. Prospective multicenter randomized control and larger sample size studies are needed to confirm the safety and efficacy of hybrid recanalization strategy.

Symptomatic post-stenting restenosis or occlusion of ICA or VA stenosis poses a challenging dilemma to neurosurgeons and neurointerventional radiologists. Symptomatic patients with post-stent restenosis or occlusion of the ICA or the VA refractory to pharmacological treatment are candidates for a revascularization procedure. The present study demonstrates that a hybrid procedure for ICA and VA post-stenting restenosis or occlusion maybe a feasible treatment strategy. A large sample size and long-term follow-up are necessary to confirm these findings.

## Conclusion

A hybrid technique that combines endarterectomy and endovascular intervention maybe a safe and feasible treatment option to recanalize in-stent restenosis or occlusion with acceptable complications.

## Data Availability Statement

The original contributions presented in the study are included in the article/supplementary materials, further inquiries can be directed to the corresponding author/s.

## Ethics Statement

The studies involving human participants were reviewed and approved by Ethics committee of Qilu hospital of Shandong University (KYLL-2020(KS)-533). The patients/participants provided their written informed consent to participate in this study. Written informed consent was obtained from the individual(s) for the publication of any potentially identifiable images or data included in this article.

## Author Contributions

DW and XL made substantial contributions to the conception and design of the work. The Operation and data acquisition were performed by PZ, TS, and MH. YW and WW performed the data analysis. CW drafted the manuscript and all of the other authors revised it critically for important intellectual content. All authors read and approved the final version to be published and they agree to be accountable for all aspects of the work in ensuring that questions related to the accuracy or integrity of any part of the work are appropriately investigated and resolved.

## Conflict of Interest

The authors declare that the research was conducted in the absence of any commercial or financial relationships that could be construed as a potential conflict of interest. The reviewer MZ declared a shared affiliation with the authors to the handling editor at time of review.

## Abbreviations

ICA: internal carotid artery
VA: vertebral artery
ISR: in-stent restenosis
ISO: in-stent occlusion
CAS: carotid artery stenting
VAS: vertebral artery stenting
DSA: digital subtraction angiography
CCA: common carotid artery
TIA: transient ischemic attacks
CEA: carotid endarterectomy
VEA: vertebral endarterectomy
CTA: CT angiography
IPH: intraplaque hemorrhage
PICA: posterior inferior cerebellar artery
PSV: Peak systolic velocity
EDV: end-diastolic velocity.
